# Bloody fluids located between the temporal muscle and targeted cerebral cortex affect the establishment of indirect collaterals in Moyamoya disease with surgical bypass: A case-control study

**DOI:** 10.3389/fneur.2022.960199

**Published:** 2022-10-26

**Authors:** Yin Li, Jun-wen Hu, Xu-chao He, Yang Cao, Xiao-bo Yu, Xiong-jie Fu, Hang Zhou, Li-bin Hu, Liang Xu, Chao-ran Xu, Yong-jie Wang, Lin Wang

**Affiliations:** ^1^Department of Neurosurgery, Second Affiliated Hospital, School of Medicine, Zhejiang University, Hangzhou, China; ^2^Clinical Research Center for Neurological Diseases of Zhejiang Province, Hangzhou, China

**Keywords:** Moyamoya disease, angiogenesis, bloody fluids, temporal muscle, digital subtraction angiography (DSA)

## Abstract

**Objective:**

Bypass yields favorable outcomes in the treatment of Moyamoya disease (MMD). Bloody fluids accumulate between the targeted cortex and the temporal muscle after surgical bypass. These fluids are handled empirically *via* subcutaneous tubes or conservative treatments. However, substances located in certain positions may adversely affect the establishment of indirect collaterals (ICs) from muscular grafts.

**Methods:**

Patients in our hospital from January 2014 to December 2019 were eligible for inclusion. Digital subtraction angiography (DSA) and radiological examinations were used during the perioperative and follow-up periods. Bloody fluid volumes were calculated using computed tomography- (CT-) based 3D Slicer software. The characteristics of bloody fluids, patient demographics, and clinical data were retrospectively analyzed.

**Results:**

In total, 110 patients underwent indirect or combined bypass with follow-up DSA. The mean age of the enrolled patients was 42.4 ± 11.8 years. Previous ischemia (*p* = 0.001), previous hemorrhage (*p* = 0.013), bloody fluid volume (*p* = 0.049), and the time of imaging (*p* = 0.081) were associated with indirect outcomes. Ordinal regression analysis confirmed that good indirect outcomes were associated with previous ischemia (*p* < 0.001) and a large bloody fluid volume (*p* = 0.013). Further subgroups based on fluid volume were significantly correlated with IC establishment (*p* = 0.030).

**Conclusions:**

A large bloody fluid volume and previous ischemic history were associated with good indirect outcomes. The presence of bloody fluids may reflect impaired degrees of muscular donors due to bipolar electrocoagulation, thus highlighting the importance of appropriate application of bipolar forceps.

## Introduction

Moyamoya disease (MMD) is a chronic cerebrovascular disease characterized by progressive steno-occlusive changes in bilateral intracranial carotid arteries and their proximal branches (1). Surgery can effectively improve cerebral hemodynamic status (2). Indirect bypass remains a high priority for pediatric patients because of its simplicity and patients' satisfactory improvements (3). Indirect bypass is also performed separately or as part of a combined bypass for treating adult patients with MMD (4). A recent study found that dual collateral establishment of direct and indirect parts occurred in only 47% of adult patients, and good indirect partial collaterals were likely to occur in patients at Suzuki stage IV or greater (5). Although strong angiogenic effects help indirect collaterals (ICs) develop toward favorable outcomes in younger patients (6), the temporal muscle takes a long time to establish valid connections with targeted brain regions. Therefore, incipient relationships must be established between the donor muscle and recipient cortices before the period when the deep temporal artery communicates further with the pial vasculature.

The initial contact between muscular grafts and brain surfaces occurs from errhysis of temporal muscles on the first post-operative day. Studies on the post-surgical accumulation of bloody fluids are lacking in evidence-based medicine. In most cases, subcutaneous tubes are empirically placed to drain residual bloody fluids. A previous study revealed that decreased levels of serum caveolin-1 and high concentrations of angiopoietin correlated with neovascularization in adults with MMD (7). Within various angiogenic factors, these fluids may affect the angiogenic interaction between the donor muscle and the arachnoid membrane. Here, we describe the role of bloody fluids in establishing ICs from the deep temporal artery and discuss the appropriate perioperative management of MMD.

## Materials and methods

### Study participants

After excluding 220 patients, 110 patients with MMD who underwent combined or indirect bypass were consecutively enrolled between January 2014 and December 2019 (Supplementary Figure 1). MMD was diagnosed as per the guidelines for MMD diagnosis and treatment published by the Research Committee on the Pathology and Treatment of Spontaneous Occlusion of the Circle of Willis (8).

Inclusion criteria were as follows: (1) MMD was definitively diagnosed *via* digital subtraction angiography (DSA). (2) Patients underwent either indirect bypass of the encephalo-duro-myo-synangiosis (EDMS) or combined bypass, of which the indirect part was EDMS. (3) Patients underwent either computed tomography (CT) or CT angiography (CTA) within 3 days post-surgery. (4) Follow-up DSA was conducted at ≥6 months post-surgery.

Exclusion criteria were as follows: (1) Neither CT nor CTA was performed within 3 days post-surgery. (2) No DSA was performed during the follow-up period. (3) Intracranial hemorrhage occurred in the acute post-operative stage.

### Surgical revascularization modalities

Surgical revascularization procedures performed in the enrolled patients were categorized as indirect or combined bypass. Criteria for surgery included the presence of transient ischemic attack or infarct, subarachnoid or intracranial hemorrhage, epilepsy, and dizziness. Indirect bypass was referred to as EDMS. Combined bypass has been described previously and consists of the superficial temporal artery (STA) to middle cerebral artery (MCA) bypass and EDMS (9). Graft patency was confirmed by intraoperative emission of indocyanine green. The temporal muscle covered the brain surface without manual splitting.

### Bloody fluid evaluation and divisions

**Figure 1:**
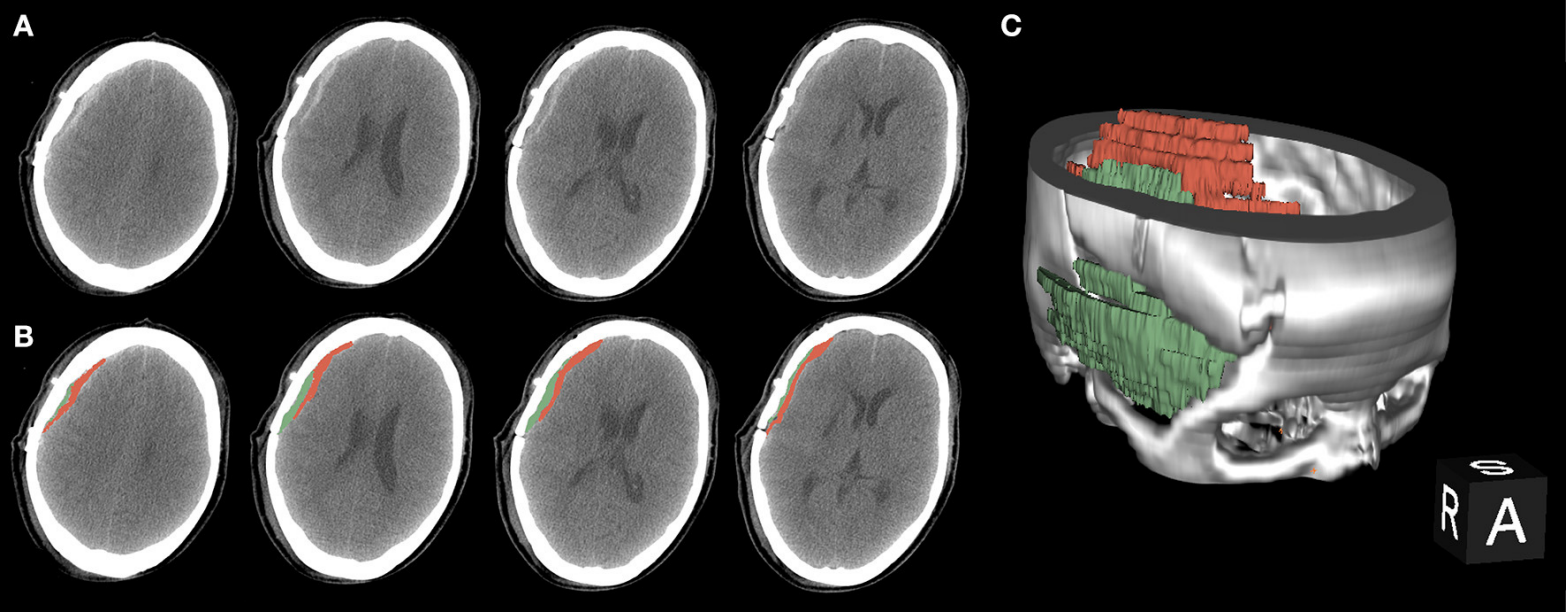
A procedure for typical bloody fluids around the operative area. **(A)** Original presentation of bloody fluids on post-operative computed tomography (CT) examinations. Black arrows indicate the separated temporal muscle. The graph shows different CT values with adjacent bloody fluids. **(B)** Bloody fluids are marked in red and the temporal muscle is marked in green using the 3D Slicer software. **(C)** The location of bloody fluids and the temporal muscle *via* 3D rebuilding.

Bloody fluid volume was calculated using 3D Slicer software (https://www.slicer.org/) after performing CT or CTA within 3 days post-surgery (Figure 1). This software enables the measurement of intracranial hemorrhage more accurately than the conventional Tada formula (10). The calculated threshold range was set as the signal range of hematomas [i.e., 50–100 Hounsfield units (HU)]. The working process went as described previously (10). The separated temporal muscle showed slight high-density signal changes on CT in the acute post-operative stage, but the signal values of the muscles did not meet the abovementioned threshold range.

Enrolled patients were grouped by bloody fluid volume as a minimum: ≤ 4 ml; small: >4– ≤ 8 ml; medium: >8– ≤ 2 ml; or large: <12 ml.

The interrater reliability of the bloody fluid volume calculation by two independent surgeons was determined and revealed that the volume and surface area of these fluids reached a near perfect agreement [interclass correlation coefficient (ICC) = 0.936 and =0.973, respectively, Table 1].

**Table 1:** The interrater reliability in the calculation involved with bloody fluids.

	**Surgeon 1**	**Surgeon 2**	***P*-value^†^**	**ICC (95% CI)**
Volume of bloody fluids, ml	7.0 ± 4.1	7.0 ± 4.4	**<0.001**	0.936 (0.908, 0.956)
Surface area, cm^2^	25.5 ± 33.1	26.4 ± 37.2	**<0.001**	0.973 (0.961, 0.982)

† *p* <0.05 are bolded.

ICC, interclass correlation coefficient.

### Outcome measures

The evaluation of collateral networks was graded using the angiographic Matsushima score (11) as follows: 0: no collaterals in the target revascularization; 1: collaterals in ≤ 1/3 of the MCA territory; 2: collaterals in 1/3–2/3 of the MCA territory; and 3: collaterals in >2/3 of the MCA territory (9). Identification of collaterals provides an overall evaluation of the compensatory area derived from either direct bypass or EDMS (Supplementary Figure 2).

This cohort was then categorized into groups based on the indirect Matsushima score as follows: (1) poor IC group: indirect Matsushima score = 0; (2) minimal IC group: indirect Matsushima score = 1; and (3) good IC group: indirect Matsushima score = 2 or 3.

### Data analysis

SPSS 24.0 (SPSS, Inc., Chicago, IL, USA) was used for statistical analysis. Differences in data between ordinal groups were compared using the Kruskal–Wallis *H*-test or Spearman's correlation as appropriate. Logistic regression analysis was conducted on factors achieving *p* < 0.10 in the univariate analysis. A *p* < 0.05 was considered statistically significant, and all tests were two-tailed.

## Results

### Demographics and clinical presentation

Of 330 consecutive patients with MMD who underwent surgery from January 2014 to December 2019, 110 were identified as meeting the inclusion criteria. Eight patients with acute intracranial hemorrhage were excluded (Supplementary Figure 1). The mean patient age was 42.4 ± 11.8 years (range 11–65 years), and 76 enrolled patients were women. Of these patients, 80.6% presented with bilateral involvement. In total, 60 patients (54.5%) experienced ischemic onset. Table 2 summarizes the other clinical characteristics.

**Table 2:** Baseline and clinical characteristics in patients with MMD who achieved surgical revascularization.

	**Patients (*n* = 110)**
Age, yrs^†^	42.4 ± 11.8 (11, 65)
Female, %	76 (69.1)
Smoking, %	20 (18.2)
Alcohol drinking, %	17 (15.4)
Hypertension, %	34 (30.9)
Diabetes, %	9 (8.2)
**Moyamoya involved**	
Bilateral	87 (79.1)
Left	12 (10.9)
Right	11 (10.0)
**Type of onset**	
Ischemia	59 (53.6)
TIA	20 (18.2)
Infarction	39 (35.5)
Hemorrhage	39 (35.5)
SAH	5 (4.5)
IVH	22 (20.0)
ICH	12 (10.9)
Epilepsy	2 (1.8)
Others	10 (9.1)
**Angiographic suzuki stage**	
I	15 (13.6)
II	14 (12.7)
III	54 (49.1)
IV	23 (20.9)
V	3 (2.7)
VI	1 (0.9)

† Values presented as mean ± standard deviation (SD) (minimum, maximum).

MMD, Moyamoya disease; TIA, transient ischemic attack; SAH, subarachnoid hemorrhage; IVH, intraventricular hemorrhage; ICH, intracranial hemorrhage.

### Surgical procedures and follow-up features

The median pre-operative disease course was 5 months (Table 3). Approximately half of the patients underwent surgery on the left side. Approximately 11 of 110 patients (10.0%) underwent indirect bypass, and 99 (90.0%) underwent combined bypass. One-fourth of the enrolled patients experienced post-operative complications, of which the most common was transient aphasia (20.0%). Four patients had post-operative seizures. Eight excluded patients underwent acute intracranial hemorrhage (Supplementary Figure 1).

**Table 3:** Surgical and follow-up features in patients with surgical revascularization.

	**Patients (*n* = 110)**
Pre-op disease period (IQR), mos	5.0 (10.0)
**Surgical type, %**	
Indirect revascularization	11 (10.0)
Combined revascularization	99 (90.0)
Surgery on the left, %	56 (50.9)
**Post-operative complications, %**	28 (25.5)
Transient neurologic deficits	
Aphasia	22 (20.0)
Weakness	4 (3.6)
Cerebral infarction	2 (1.8)
Epilepsy	4 (3.6)
Length of stay (IQR), days	15.0 (5.0)
Follow-Up period (IQR), mos	11.5 (4.5)
**Indirect matsushima score, %**	
0	42 (38.2)
1	50 (45.5)
2	17 (15.5)
3	1 (0.9)
**Direct matsushima score, %**	
0	0 (0.0)
1	34 (34.3)
2	35 (45.5)
3	20 (20.2)

Pre-op, pre-operative; IQR, interquartile range.

The radiographic follow-up period was 11.5 months. Angiographic angiogenesis was assessed for both indirect and direct parts, of which 18 of 110 (16.4%) patients had indirect scores ≥2, and 65.7% (55/99) had direct scores ≥2 on the Matsushima angiogenesis classification. Direct scores were more likely to grade favorably than indirect scores for all enrolled patients.

### Factors related to establishing ICs

Patients were further categorized into poor, minimal, and good IC groups, as described in Section Outcome measures. Demographic characteristics were consistent and did not statistically differ among the groups (Table 4). In the good IC group, 88.9% experienced previous ischemic onset, which was positively correlated with indirect outcomes (*p* = 0.001). Conversely, initial hemorrhage was more common in the poor IC group than in the other two groups (*p* = 0.013). Surgery on the left side was less common in the good IC group than in the poor and minimal IC groups (38.9 vs. 61.9 and 46.0%, respectively, *p* = 0.172). Bloody fluid volume was significantly correlated with good indirect outcomes (*p* = 0.049). The average time points for post-operative imaging were similar, occurring ~2 days post-surgery. The mean follow-up periods for all groups exceeded 12 months.

**Table 4:** Factors related to the generation of indirect collaterals in MMDs after bypass.

	**Univariate analysis**				**Ordinal regression analysis**		
	**Poor ICs (*n* = 42)**	**Minimal ICs (*n* = 50)**	**Good ICs (*n* = 18)**	***P*-value^†^**	**OR**	**95% CI**	***P*-value^†^**
Age, yrs	41.7 ± 13.6	42.6 ± 11.7	43.1 ± 6.9	0.679^‡^			
Female (%)	33 (78.6)	30 (60.0)	13 (72.2)	0.153^§^			
Smoking (%)	5 (11.9)	12 (24.0)	3 (16.7)	0.323^§^			
Bilateral involved	33 (78.6)	39 (78.0)	15 (83.3)	0.888^§^			
Previous ischemia	15 (35.7)	28 (56.0)	16 (88.9)	**0.001** ^§^	4.724	2.161–10.327	**<0.001**
Previous hemorrhage	21 (50.0)	16 (32.0)	2 (11.1)	**0.013** ^§^			0.870
Initial suzuki stage (%)				0.157^‡^			
I	9 (21.4)	6 (12.0)	0 (0.0)				
II	5 (11.9)	8 (16.0)	1 (7.1)				
III	19 (45.2)	22 (44.0)	13 (72.2)				
IV	6 (14.3)	13 (26.0)	4 (22.2)				
V	3 (7.1)	0 (0.0)	0 (0.0)				
VI	0 (0.0)	1 (2.0)	0 (0.0)				
Surgery on the left (%)	26 (61.9)	23 (46.0)	7 (38.9)	0.172^§^			
**Features of post-op blood fluids**							
Volume, ml	5.9 ± 3.4	7.6 ± 4.5	7.9 ± 4.4	**0.049** ^‡^	1.122	1.025–1.228	**0.013**
Surface area, cm^2^	27.5 ± 38.1	19.3 ± 25.6	40.9 ± 45.6	0.333^‡^			
Time of imaging, days	1.9 ± 1.0	2.0 ± 1.0	2.4 ± 1.0	0.081^‡^			0.159
Post-operative complications, %	10 (23.8)	10 (20.0)	8 (44.4)	0.121^§^			
Follow-up period, mos	12.0 ± 5.9	14.3 ± 13.1	13.5± 5.7	0.221^‡^			

^†^*p* < 0.05 are bolded.

^‡^Spearman's correlation.

^§^Kruskal–Wallis H-test.

ICs, indirect collaterals; Ref, reference category; Post-op, post-operative.

**Figure 2:**
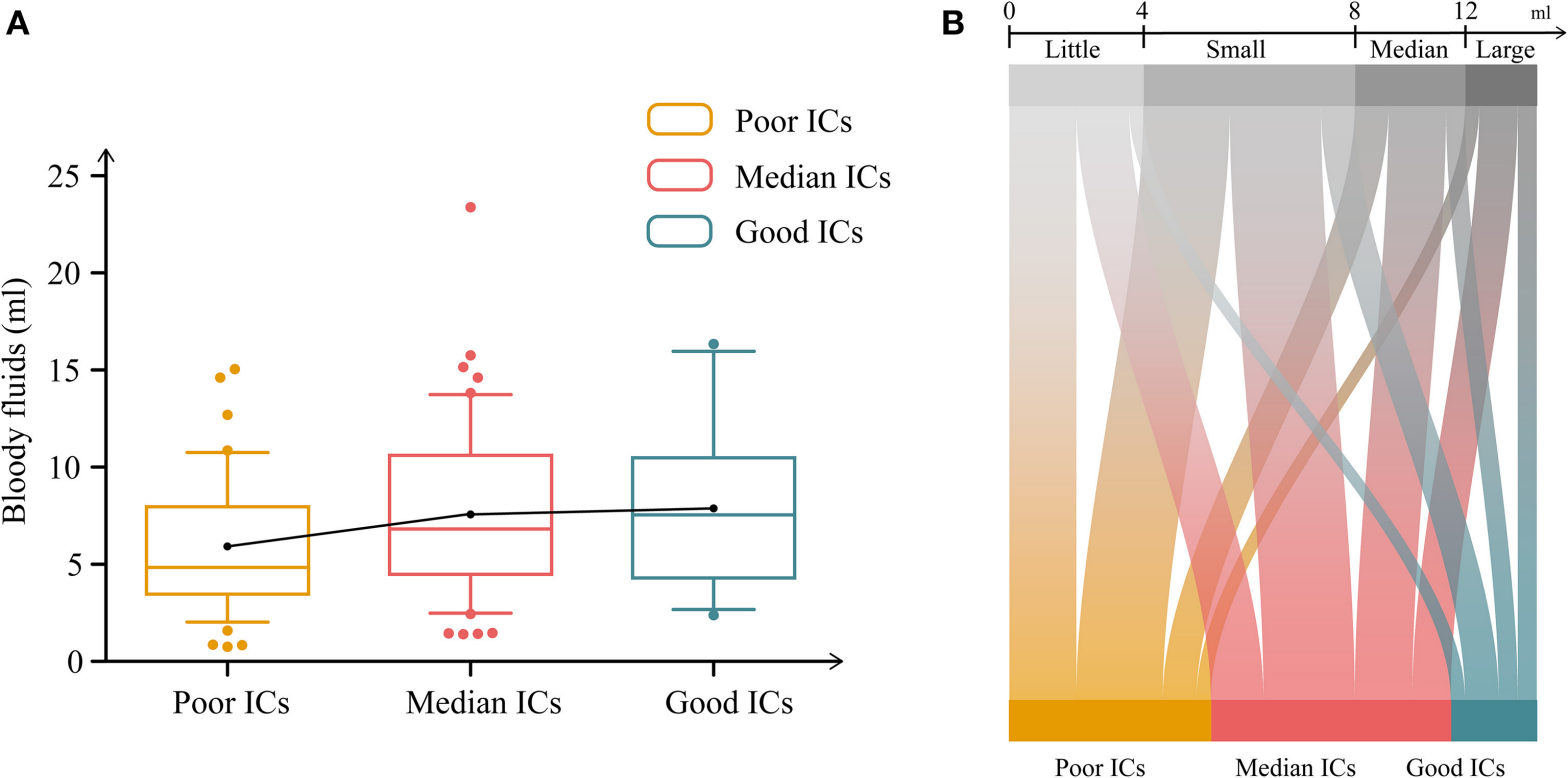
Relationship between bloody fluid volume and indirect outcomes. **(A)** Differences in bloody fluid volumes among the groups categorized by indirect collaterals (ICs). **(B)** Differences in ICs among post-operative bloody fluid volumes. Minimal bloody fluid volume: <4 ml; small fluid volume: 4–8 ml; medium fluid volume: 8–12 ml; and large fluid volume >12 ml.

All possible factors were included in the ordinal regression analysis, in which the parallel line test met the standard (Supplementary Table 1). Previous ischemia and bloody fluid volume were independent factors, which were related to indirect outcomes (Table 4). Imaging time was a potential confounding factor in the regression analysis, and no statistically significant differences were observed (*p* = 0.159). Increases in bloody fluid volume were correlated with good ICs [odds ratio (OR): 1.122, 95% confidence interval (CI): 1.025–1.228, *p* = 0.013, Table 4, Figure 2A]. Patients with previous ischemic histories were prone to favorable IC outcomes during a follow-up (OR: 4.724, 95% CI: 2.161–10.327, *p* < 0.001). Patients with hemorrhage at onset were more likely to have poor IC outcomes (*p* = 0.130); however, the ordinal regression analysis showed no statistical differences (*p* = 0.870).

### Bloody fluid features

We further explored clinical and follow-up differences among patients divided by bloody fluid volume grading (Section 2.3). In total, 28 patients had a minimal bloody fluid volume, 44 had a small volume, 23 had a medium volume, and 15 had a large volume. Table 5 compares the patient demographics, clinical features, surgeries, and follow-up characteristics. Only the IC grades differed significantly. The following subgroup analysis of initial onset was conducted in MMD based on the classification of post-operative bloody fluids (Supplementary Table 2). In the subgroup of ischemia, surgery on the left presented significant differences (*p* = 0.018). In contrast, post-operative bloody fluid volume showed statistical significance in the subgroup of hemorrhage (*p* = 0.009). Good IC grades were correlated with a large bloody fluid volume (*p* = 0.030; Figure 2B). Conversely, the direct Matsushima score was not associated with post-operative bloody fluid volume and did not significantly differ among groups (*p* = 0.550). Postoperative imaging times did not significantly differ among the bloody fluid volume levels (*p* = 0.376).

**Table 5:** Comparison of clinical features, surgical and follow-up characteristics in MMDs based on the classification of post-operative bloody fluids.

	**Little (*n* = 28)**	**Small (*n* = 44)**	**Median (*n* = 23)**	**Large (*n* = 15)**	***P*-value^†^**
Age, yrs	41.4 ± 13.8	42.9 ± 11.4	40.1 ± 11.5	45.9 ± 8.9	0.864^‡^
Female (%)	20 (71.4)	33 (75.0)	15 (65.2)	8 (53.3)	0.445^§^
Smoking (%)	3 (10.7)	8 (18.2)	5 (21.7)	4 (26.7)	0.582^§^
Bilateral involved	23 (82.1)	35 (79.5)	17 (73.9)	12 (80.0)	0.910^§^
Type of onset					0.536^§^
Ischemia	16 (57.1)	23 (52.3)	13 (56.5)	1 (6.7)	
Hemorrhage	8 (28.6)	17 (38.6)	7 (30.4)	7 (46.7)	
Others	4 (14.3)	4 (9.1)	3 (13.0)	7 (46.7)	
Initial suzuki stage (%)					0.204^‡^
I	6 (21.4)	7 (15.9)	2 (8.7)	0 (0.0)	
II	2 (7.1)	5 (11.4)	5 (21.7)	2 (13.3)	
III	14 (50.0)	23 (52.3)	10 (43.5)	7 (46.7)	
IV	4 (14.3)	8 (18.2)	5 (21.7)	6 (40.0)	
V	2 (7.1)	0 (0.0)	1 (4.3)	0 (0.0)	
VI	0 (0.0)	1 (2.3)	0 (0.0)	0 (0.0)	
Surgery on the left (%)	14 (50.0)	22 (50.0)	10 (43.5)	10 (66.7)	0.572^§^
Surface area, cm^2^					
Time of post-op imaging, days	2.0± 1.0	2.0 ± 1.1	2. ± 1.0	2.270 ± 0.884	0.376^‡^
Post-operative complications, %	6 (21.4)	8 (18.2)	9 (39.1)	5 (33.3)	0.244^§^
Follow-up period, mos	11.2 ± 3.9	15.5 ± 14.1	13.1 ± 6.4	11.1 ± 3.8	0.838^‡^
ICs grades, %					**0.030** ^§^
Poor	14 (50.0)	18 (40.9)	7 (30.4)	3 (20.0)	
Minimal	11 (39.3)	19 (43.2)	12 (52.2)	8 (53.3)	
Good	3 (10.7)	7 (15.9)	4 (17.4)	4 (26.7)	
Direct matsushima score, %					0.550^‡^
1	8 (30.8)	11 (28.2)	10 (47.6)	5 (38.5)	
2	13 (50.0)	20 (51.3)	8 (38.1)	4 (30.8)	
3	5 (19.2)	8 (20.5)	3 (14.3)	4 (30.8)	

^†^*p* < 0.05 are highlighted in bold.

^‡^Spearman's correlation.

^§^Kruskal–Wallis H-test.

Post-op, post-operative; ICs, indirect collaterals.

## Discussion

Single and combined bypass are used to treat MMD, and both yield satisfactory outcomes. Studies have explored the long-term patency of anastomotic sites or factors related to direct partial outcomes (12–14). Previous studies have confirmed the association between younger age and indirect partial outcomes (6). However, when establishing collateral networks among patients with MMD, the combination of STA-MCA bypass and EDMS may have opposite outcomes and yield opposite results. Furthermore, collateral cerebral development of single EDMS varies among patients. Here, we found that a large post-operative bloody fluid volume and previous ischemia were associated with good indirect outcomes.

### Initial type of onset

The proportions of patients with previous ischemia increased from the poor to the good IC subgroups, with a decreasing trend in the initial hemorrhage (Table 4). After entering the ordinal regression, previous ischemia was a potential factor for good indirect outcomes. A multicenter study revealed that intracranial hemorrhage was an independent predictor of poor neovascularization for patients with MMD undergoing indirect bypass (15). Subgroup analysis showed that bloody fluid volume was related to ICs in the hemorrhagic group (Supplementary Table 2). In contrast to those with hemorrhagic MMD, patients with ischemic onset exhibited favorable indirect outcomes. The hemodynamic status in ischemic MMD remained stable, and the pial vasculature in these patients did not suffer any hemorrhagic strikes. This may lay a foundation for a preliminary connection between the temporal muscle and the recipient vessels through the pial vasculature, further forming ICs from the deep temporal artery.

### Post-operative bloody fluid volume

**Figure 3:**
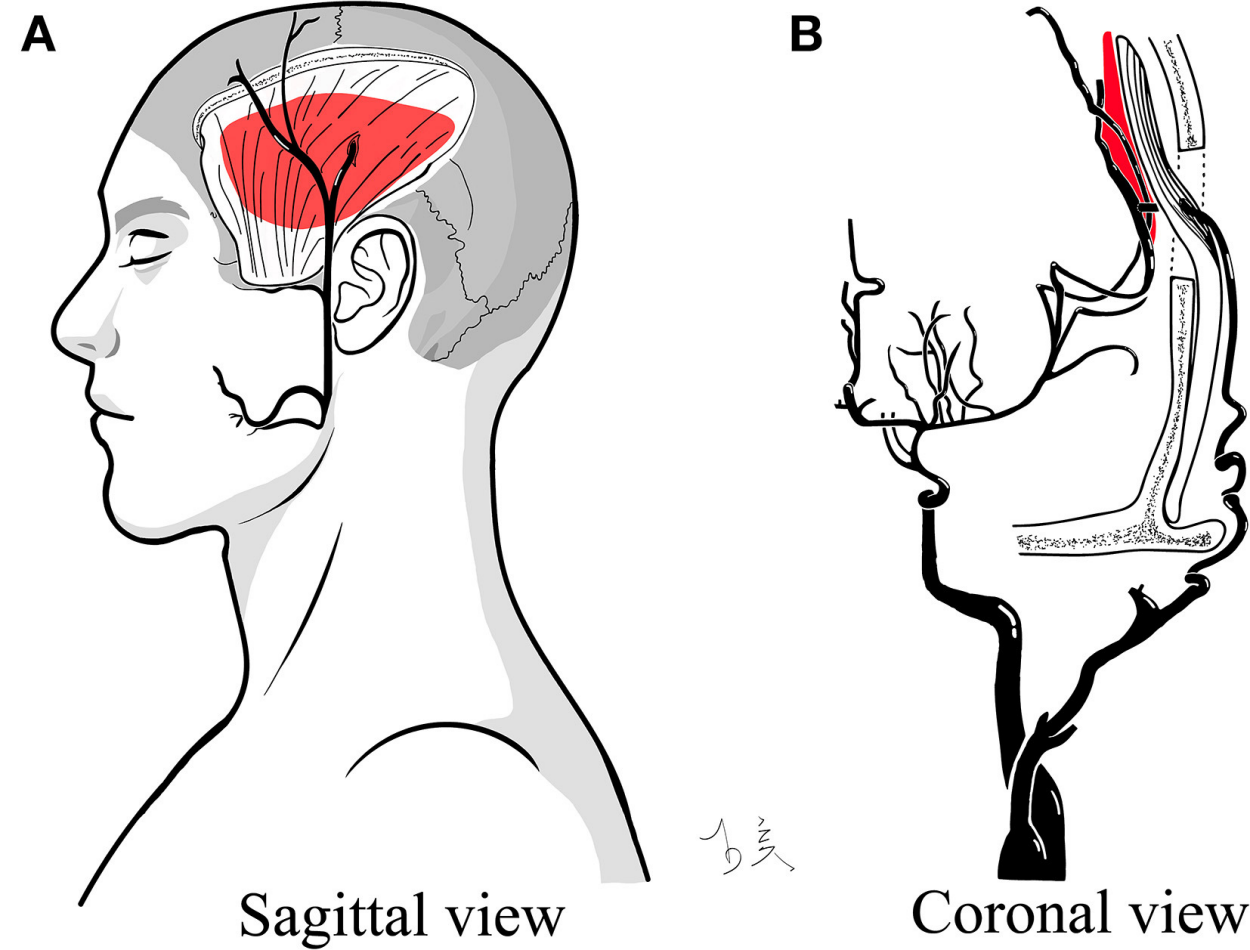
Illustration of bloody fluids between the separated temporal muscle and targeted cortex. The red mass indicates post-operative bloody fluids and their specific positions. **(A)** Sagittal view. **(B)** Coronal view.

Postoperative bloody fluid volume was significantly correlated with indirect partial outcomes, which was supported by subsequent subgroup analysis (Table 4, Figure 2B). Unlike STA-MCA bypass, which reverses the impaired cerebral hemodynamic status immediately after surgery, it takes a long period for the temporal muscle to effectively associate with cortical regions *via* the leptomeningeal vessels (16). Hence, breaking down the barriers between the donor grafts and recipient arteries will help to further develop ICs. The specific location of bloody fluids greatly affects the contact between the separated temporal muscle and the pial vasculature (Figure 3).

Large volumes between the two layers may contain more angiogenesis-related cytokines, in contrast to the small volumes of bloody fluids. The specific allele of vascular endothelial growth factor (VEGF) is associated with the poor collateral formation in MMD (17). Previous studies confirmed that the VEGF levels were nearly identical to those in healthy cohorts, confirming an active neovascularization state in patients with MMD (7) despite decreased serum caveolin-1 concentrations associated with negative arterial remodeling (18). Increased serum angiopoietin-2 levels have been described (19), and angiopoietins contained in these fluids may facilitate vascular responses that regulate angiogenesis *via* an autocrine release in patients with MMD (20). Additionally, surgical bypass can elevate VEGF-A_165_ a/b ratios in MMD and intracranial atherosclerotic disease (21). These factors associated with neovascularization may be involved in initially establishing ICs, which contain these fluids between donor grafts and recipient encephalic regions.

Another hypothesis that may better account for this is that bloody fluid volume may be the epitome of donor muscular status. Bloody fluids inevitably accumulate after surgery, mostly because of errhysis of muscular grafts or from the outflow from anastomotic sites. During indirect or combined bypass, the temporal muscle is stripped from the skull and handled carefully *via* electrocoagulation, which may cause microvascular occlusion of the inner muscular side (22). Excessive application of bipolar forceps to the inner face of the separated muscle reduced the errhysis to a small volume in the first few hours after surgery. Conversely, a large volume of bloody fluids served as a possible indicator of well-preserved muscular grafts escaping immoderate electrocoagulation and implied a beneficial microvascular environment for collaterals through pia meningeal vessels in recipient encephalic regions. These fluid volumes may reflect the impaired damage caused by electrocoagulation, suggesting that proper use of bipolar forceps is important when dealing with temporal muscles without severe arterial bleeding.

The large distance associated with large bloody fluid volumes may have blocked interactions between this donor muscle and recipient regions. Unfortunately, these results were not consistent with this trend. Medium and large volume of bloody fluids had similar results in the proportions of indirect outcomes (30.4 vs. 20.0, 52.2 vs. 53.3, and 17.4 vs. 26.7%; Table 5, Figure 2B). The largest volume was 23.4 ml from a patient in the minimal IC group. This may indicate that the inflection is reversed from positive to negative effects in ICs. The smallest size calculated by our software was 0.8 ml. The threshold range calculated by the software neglected the diluted portion mixed with subdural fluids, giving the illusion of minimal bloody fluids.

This study had several limitations. First, the threshold range calculated by the software may have neglected the dilute portion that is mixed with the subdural fluids, giving the illusion of minimal bloody fluids because the smallest size obtained was 0.761 ml. Second, operational factors, such as bipolar electrocoagulation frequency in the temporal muscle and integrity of the separated muscular grafts, could not be analyzed and might have resulted in a lack of evaluation criteria and data. Finally, there may be a selection bias between enrolled and excluded patients at baseline characteristics. Some cytokines possibly associated with collateral improvements in patients should also be confirmed. The conclusion requires further prospective studies with a larger MMD cohort to determine the critical point and mechanisms of MMD.

## Conclusions

Bloody fluids between the temporal muscle and targeted brain regions are familiar manifestations in patients with MMD after surgery. In this study, the large volume of these fluids and previous ischemic history were associated with good indirect outcomes. The presence of bloody fluids reliably reflects muscle donor impairment due to bipolar electrocoagulation, which may indicate the active status of the temporal muscle, and highlights the need for appropriate application of the bipolar forceps. These findings also suggest that inadequate drainage may be acceptable, as this has occurred in patients undergoing EDMS and combined bypass. In summary, bloody fluids may have positive effects on long-term indirect outcomes.

## Data availability statement

The raw data supporting the conclusions of this article will be made available by the authors, without undue reservation.

## Ethics statement

The studies involving human participants were reviewed and approved by the Local Ethics Committee of the Second Affiliated Hospital of School of Medicine, Zhejiang University. Written informed consent to participate in this study was provided by the participants' legal guardian/next of kin.

## Author contributions

Material preparation, data collection, and analysis were performed by YL and J-WH. The first draft of the manuscript was written by YL. All authors commented on previous versions of the manuscript, contributed to the study conception and design, read, and approved the final manuscript.

## Funding

This work was supported by the National Science Foundation of China (Grant Nos. 81870910 and 82171271) and Natural Science Foundation of Zhejiang Province (LQ19H160039).

## Conflict of interest

The authors declare that the research was conducted in the absence of any commercial or financial relationships that could be construed as a potential conflict of interest.

## Publisher's note

All claims expressed in this article are solely those of the authors and do not necessarily represent those of their affiliated organizations, or those of the publisher, the editors and the reviewers. Any product that may be evaluated in this article, or claim that may be made by its manufacturer, is not guaranteed or endorsed by the publisher.

## Abbreviations

CT: computed tomography
DSA: digital subtraction angiography
EDMS: encephalo-duro-myo-synangiosis
ICs: indirect collaterals
MCA: middle cerebral artery
MMD: Moyamoya disease
STA: superficial temporal artery
VEGF: vascular endothelial growth factor.
