# Supplementary material for: Bloody fluids located between the temporal muscle and targeted cerebral cortex affect the establishment of indirect collaterals in Moyamoya disease with surgical bypass: A case-control study
Source: Front Neurol. 2022 Oct 26;13:960199. doi: 10.3389/fneur.2022.960199 (PMC9644190; doi:10.3389/fneur.2022.960199)
Supplement: Supplementary file 1 [file Data_Sheet_1.docx]

**Table S1 The parallel line test of the ordinal regression analysis**

| Model | -2 log likelihood | Chi-square | Degree of freedom | Significance |
| --- | --- | --- | --- | --- |
| Original hypothesis | 195.231 |  |  |  |
| Regular | 190.751 | 4.480 | 5 | 0.483 |





**Figure S1**: Flow diagram for the study selection process.


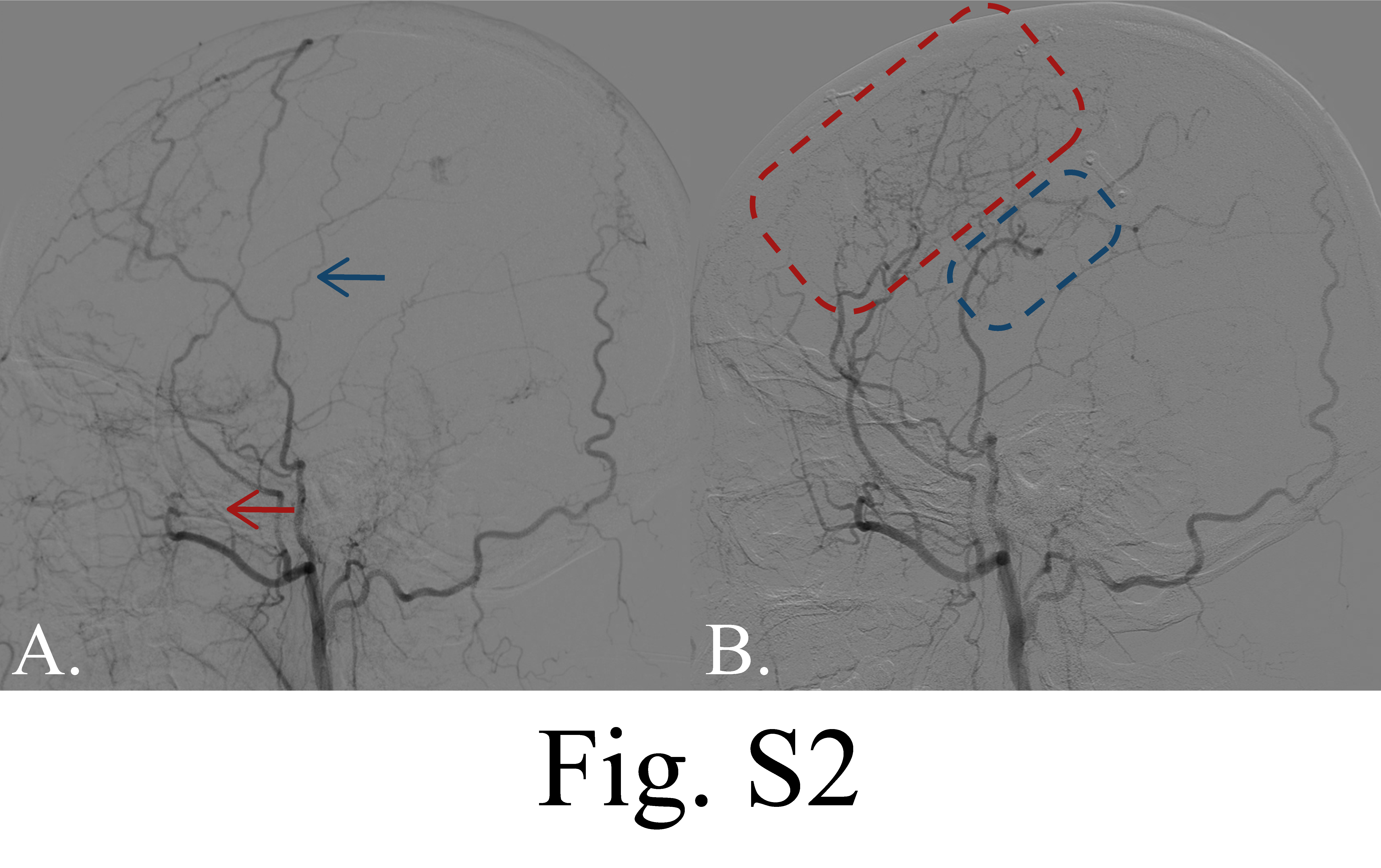


**Figure S2:** The identification of follow-up collaterals from direct or indirect revascularization.

A. indicated that deep temporal artery (DTA) which supplied the temporal muscle was marked by the red arrow and superficial temporal artery (STA) marked by the blue arrow in the lateral view of preoperative digital subtraction angiography (DSA). B. presented the follow-up establishment of collaterals on DSA. The red circle indicated collaterals from the temporal muscle (encephalo-duro-myo-synangiosis) and the blue circle indicated collaterals from STA (STA-MCA anastomosis).

**Table S2** The subgroup analysis of initial onset in MMDs based on the classification of postoperative bloody fluids

|  |  | Poor ICs | Minimal ICs | Good ICs | *P* value ^†^ |
| --- | --- | --- | --- | --- | --- |
| Ischemic subgroup | Patients | 15 | 28 | 16 |  |
|  | Age, yrs | 43.5 ± 15.4 | 41.7 ± 12.2 | 42.8 ± 7.3 | 0.605 ^‡^ |
|  | Female (%) | 11 (73.3) | 16 (57.1) | 11 (68.8) | 0.820 ^§^ |
|  | Smoking (%) | 2 (13.3) | 5 (17.9) | 3 (18.8) | 0.698 ^§^ |
|  | Bilateral involved | 13 (86.7) | 24 (85.7) | 13 (81.3) | 0.677 ^§^ |
|  | Initial Suzuki stage (%) |  |  |  | 0.551 ^‡^ |
|  | Ⅰ | 2 (13.3) | 4 (14.3) | 0 (0.0) |  |
|  | Ⅱ | 3 (20.0) | 4 (14.3) | 0 (0.0) |  |
|  | Ⅲ | 5 (33.3) | 12 (42.9) | 12 (75.0) |  |
|  | Ⅳ | 5 (33.3) | 8 (28.6) | 4 25.0) |  |
|  | Ⅴ | 0 (0.0) | 0 (0.0) | 0 (0.0) |  |
|  | Ⅵ | 0 (0.0) | 0 (0.0) | 0 (0.0) |  |
|  | Surgery on the left (%) | 12 (80.0) | 14 (50.0) | 6 (37.5) | **0.018** ^§^ |
|  | Features of postop blood fluids |  |  |  |  |
|  | Volume, ml | 5.7 ± 3.6 | 6.7 ± 4.0 | 7.3 ± 4.2 | 0.637 ^‡^ |
|  | Surface area, cm^2^ | 19.8 ± 22.8 | 18.7 ± 25.6 | 35.7 ± 38.9 | 0.447 ^‡^ |
|  | Time of imaging, days | 1.9 ± 0.7 | 2.0 ± 0.9 | 2.4 ± 1.0 | 0.264 ^‡^ |
|  | Postoperative complications, % | 3 (20.0) | 6 (21.4) | 7 (43.8) | 0.135 ^‡^ |
|  | Follow-up period, mos | 11.5 ± 3.9 | 14.7 ± 16.0 | 13.8± 5.9 | 0.681 ^‡^ |
| Hemorrhagic subgroup | Patients | 21 | 16 | 2 |  |
|  | Age, yrs | 39.2 ± 13.3 | 44.1 ± 12.2 | 45.5 ± 2.1 | 0.598 ^‡^ |
|  | Female (%) | 17 (81.0) | 10 (62.5) | 2 (100.0) | 0.450 ^§^ |
|  | Smoking (%) | 3 (14.3) | 5 (31.3) | 0 (0.0) | 0.425 ^§^ |
|  | Bilateral involved | 16 (76.2) | 13 (81.3) | 2 (100.0) | 0.522 ^§^ |
|  | Initial Suzuki stage (%) |  |  |  | 0.490 ^‡^ |
|  | Ⅰ | 4 (19.0) | 2 (12.5) | 0 (0.0) |  |
|  | Ⅱ | 1 (4.8) | 1 (6.3) | 1 (50.0) |  |
|  | Ⅲ | 12 (57.1) | 7 (43.8) | 1 (50.0) |  |
|  | Ⅳ | 1 (4.8) | 5 (31.3) | 0 (0.0) |  |
|  | Ⅴ | 3 (14.3) | 0 (0.0) | 0 (0.0) |  |
|  | Ⅵ | 0 (0.0) | 1 (6.3) | 0 (0.0) |  |
|  | Surgery on the left (%) | 11 (52.4) | 7 (43.8) | 1 (50.0) | 0.661 ^§^ |
|  | Features of postop blood fluids |  |  |  |  |
|  | Volume, ml | 6.0 ± 3.1 | 9.5 ± 5.2 | 12.9 ± 0.7 | **0.009** ^‡^ |
|  | Surface area, cm^2^ | 31.7 ± 45.0 | 19.0 ± 27.2 | 82.4 ± 93.3 | 0.333 ^‡^ |
|  | Time of imaging, days | 2.0 ± 1.1 | 1.8 ± 1.1 | 2.5 ± 0.7 | 0.526 ^‡^ |
|  | Postoperative complications, % | 4 (19.0) | 4 (25.0) | 1 (50.0) | 0.445 ^‡^ |
|  | Follow-up period, mos | 12.8 ± 7.5 | 14.9 ± 9.1 | 10.7± 4.3 | 0.572 ^‡^ |

^†^ (*P* <0.05) are bolded;

^‡^: Spearman correlation;

^§^: Kruskal-Wallis H test;

^‡^: Spearman correlation;

ICs, Indirect Collaterals;

Ref, Reference category;

Postop, postoperative.
